# Load frequency stabilization of distinct hybrid conventional and renewable power systems incorporated with electrical vehicles and capacitive energy storage

**DOI:** 10.1038/s41598-024-60028-3

**Published:** 2024-04-24

**Authors:** Amil Daraz, Hasan Alrajhi, Abdul Basit, Abdul Rahman Afzal, Ahmed N. M. Alahmadi, Irfan Ahmed Khan

**Affiliations:** 1 College of Information Science and Electronic Engineering, Zhejiang University, Hangzhou, China; 2 Electrical Engineering Department, College of Engineering and Islamic Architecture, Umm Al-Qura University, Makkah, 21955 Saudi Arabia; 3 Department of Industrial Engineering, University of Business and Technology (UBT) University, Jeddah, Saudi Arabia; 4 Department of Electrical Engineering, Faculty of Engineering, Universiti Malaya, Kuala Lumpur, Malaysia; 5 MEU Research Unit, Middle East University, Amman, Jordan

**Keywords:** Load frequency regulation, Fractional order controller, Renewable energy resources, Heuristic algorithm, Squid game optimizer, Interconnected power system, Energy science and technology, Engineering, Mathematics and computing

## Abstract

Maintaining a power balance between generation and demand is generally acknowledged as being essential to maintaining a system frequency within reasonable bounds. This is especially important for linked renewable-based hybrid power systems (HPS), where disruptions are more likely to occur. This paper suggests a prominent modified “Fractional order-proportional-integral with double derivative (FOPIDD2) controller” as an innovative HPS controller in order to navigate these obstacles. The recommended control approach has been validated in power systems including wind, reheat thermal, solar, and hydro generating, as well as capacitive energy storage and electric vehicle. The improved controller’s performance is evaluated by comparing it to regular FOPID, PID, and PIDD2 controllers. Furthermore, the gains of the newly structured FOPIDD2 controller are optimized using a newly intended algorithm terms as squid game optimizer (SGO). The controller’s performance is compared to benchmarks such as the grey wolf optimizer (GWO) and jellyfish search optimization. By comparing performance characteristics such as maximum frequency undershoot/overshoot, and steadying time, the SGO-FOPIDD2 controller outperforms the other techniques. The suggested SGO optimized FOPIDD2 controller was analyzed and validated for its ability to withstand the influence of power system parameter uncertainties under various loading scenarios and situations. Without any complicated design, the results show that the new controller can work steadily and regulate frequency with an appropriate controller coefficient.

## Introduction

A sharp increase in demand, coupled with greater exhaustion of fossil fuels, is driving the use of unconventional resources in today’s electricity grid. The transition of an energy system with low emissions and its adoption of policies has been detailed in references^1,2^. In this situation, a hybrid power system combined with non-conventional supplies is thought to be the cheapest option due to its energy security, on-site allocated energy supply and small-scale analysis. However, mismatches between net production and demand are common in hybrid power systems, causing frequency and power fluctuations. To evade this difficulty in the current power system modeled, automatic load frequency regulator serves an important role in preserving the equilibrium between consumption and production through system frequency management and power allocation. The frequency variation is mostly governed by both main and auxiliary controls^3^. Secondary control plays a vital role in regulating frequency after significant deviations or incidents while a governor has a control procedure that allows it to modify speed and frequency in elementary control^4,5^. As a result, secondary control is vital in regulating frequency after significant deviations or incidents^4^. Renewable energy storages (RESs) technology yields an abundance of benefits, including the reduction of greenhouse gas emissions and the improvement of sustainability of the energy system, despite their draw backs of lower inertia and intermittent behavior^6,7^.

### Literature study

Considerable support has been made by the researchers in order to tackle the frequency regulation issue in the PS. For instance, the authors has examined Load Frequency Control (LFC) in single-area systems (referenced as^8,9^), deregulated energy grids (referenced as^10,11^) , and multi-zone systems with non-linearities (referenced as^12–14^) To manage load frequency in power systems, numerous control mechanisms have been implemented, including robust sliding mode controllers^15^, model predictive control reference in^16^, linear-matrix inequality^17^, artificial intelligence-based LFC^18^, resilient control methodologies^19^, data-driven controllers^20^, and robust virtual inertia control^21^ and fuzzy logic control (FLC) referenced as^22^. Historically, the PID controller has been the predominant choice for regulating the frequency of interconnected power systems owing to its straightforwardness and economical nature. However, adjusting the PID controller to account for nonlinear characteristics and system interruptions is difficult and necessitates considerable experimentation in order to ascertain the most effective PID values. There has been a surge in interest regarding the utilization of fractional order controllers for power optimization control due to recent developments in computing power. Significantly, secondary LFCs have been implemented using FOPID and its modifications in interconnected two-region power systems^23–25^. Additionally, cascaded controller types^26–28^ have been implemented in an effort to improve frequency stability. Modern studies^29,30^ investigate a dual-controller integration strategy, with a particular focus on the PIDD2 controller framework, which was introduced in^31^ to mitigate frequency fluctuations in a two-area coupled power system. Furthermore, for frequency adaptation of the system, the literature^32,33^ suggests the integral-tilt derivative (ITD) and FOI-TD controllers; of these, the ID-T controller exhibits greater frequency efficacy than the TID controller^34^. Fractional calculus has been extensively applied to improve upon traditional PID controllers. In numerous engineering applications, including LFC systems, FOPID and PIDD2 controllers have proven to be advantageous. In order to enhance the transient and dynamic performance of LFCs, we present an innovative FOPIDD2 controller. This controller forms a hybrid framework by integrating PIDD2 and fractional calculus.

Aforementioned research illustrates that the selection of the controller type is equally as crucial as the selection of the controller parameters. The implementation of evolutionary optimization for controller parameter optimization has resulted in a significant enhancement of the frequency stability issue. Sophisticated new techniques are implemented in order to optimize the controller parameters and surmount the intricacy of the control schemes. As an illustration, the authors implemented the following algorithms: self-tuned algorithm (STA)^35^, Bull–Lion Optimization (BLO)^36^, Differential Evolution based PI regulator for automatic generation control^37^,chaos game optimization (CGO)^38^, Krill herd algorithm for AGC of multi-region non-linear power system^39^, improved fitness dependent algorithm based tuned modified FOPID controller employed in deregulated environment^40^, modified multiverse optimizer^41^, and Sunflower optimization algorithm (BOA)^42^. The authors in^43^ have been using pathfinder optimizer algorithm (PFA) to balance load power demand employing FOTID regulator. According to research^44^, the I/PI/PID controller was surpassed by the TID with filter (TIDF) configured with DE. In a similar vein, the efficacy of the modified tilt integral derivative controller optimized with the water cycle algorithm (WCA) surpassed that of PID/TID controllers^45^.

The present study introduces a recent and strong metaheuristic algorithm called the Squid Game Optimizer (SGO) that draws inspiration from the fundamental principles of a traditional Korean sport in order to determine the optimal parameters for the proposed FOPIDD2 controller. Assailants aim to achieve their objective during the cephalopod game, whereas teams compete to eliminate one another. It is generally executed on expansive, unrestricted areas lacking any predetermined boundaries regarding scope or dimensions. Historical records indicate that the court for this sport is typically squid-shaped and seems to be half the size of a normal basketball court. The mathematical formulation of this approach is initially constructed through the random selection of optimal candidate solutions and an initialization method. Solution candidates engage in combat with defensive players in two groups, instigating a rematch that involves arbitrary movement in the opposite direction. The position update procedure has been finalized, and the champion states of the players on opposing factions are utilized to generate the current position vectors. The estimates for these states are derived from the cost function. The performance of the presented SGO algorithm is evaluated using twenty-five (25) unrestricted mathematical assessment functions in addition to six other commonly employed metaheuristics for assessment. In addition, the capability of the proposed SGO is assessed through the utilization of sophisticated real-world challenges on the most recent CEC-2020^46^. The SGO exhibits exceptional performance in addressing these thought-provoking optimization issues.

Furthermore, an extensive review of the relevant literature leads to the fundamental conclusion that LFC methods, including FLC, model predictive control (MPC) and H-infinite approaches achieve the desired performance despite a protracted setup process and numerous design flaws. Furthermore, traditional PD, PI, and PID controllers encounter difficulties when confronted with system uncertainty. In a number of earlier works, the influence of boundary fluctuations and system nonlinearities on robustness evaluations was inadequately investigated. The majority of prior assessments failed to account for the substantial incorporation of renewable energy sources, despite the inclusion of nonlinear system uncertainties and immediate fluctuations in demand, without modifying system parameters.

### Contribution of the paper

FOPIDD2 controller is presented in this study with the objective of improving the frequency stability of the system while accounting for disturbances caused by renewable energy sources. In accordance with the tenets of the SGO, the parameters of the FOPIDD2 controller that has been proposed have been adjusted to guarantee frequency stability and system performance under atypical circumstances. The principal contributions of this paper, relative to previous investigations on analogous subjects, encompass:

- Incorporating an efficient FOPIDD2 controller into dual zone coupled power systems that incorporate Capacitive Energy Storage (CES), Renewable Energy Sources (RES), and Electric Vehicles (EVs) in order to enhance frequency stability.
- Introducing a strong robust algorithm, the Squid Game Optimizer (SGO), to fine-tune the parameters of the presented FOPIDD2 controller.
- Validating the superiority of the proposed FOPIDD2 controller over existing PIDD2/PID/ FOPID controllers.
- Demonstrating the effectiveness of the Squid Game Optimizer in comparison to other contemporary algorithms, such as the Grey Wolf Optimizer (GWO) and Jellyfish Swarm Optimization (JSO).
- Assessing the resilience of the suggested controller in the presence of significant fluctuations in all system parameters, and random load disturbances.

## Investigation of hybrid power systems including non-linearities

**Figure 1:**
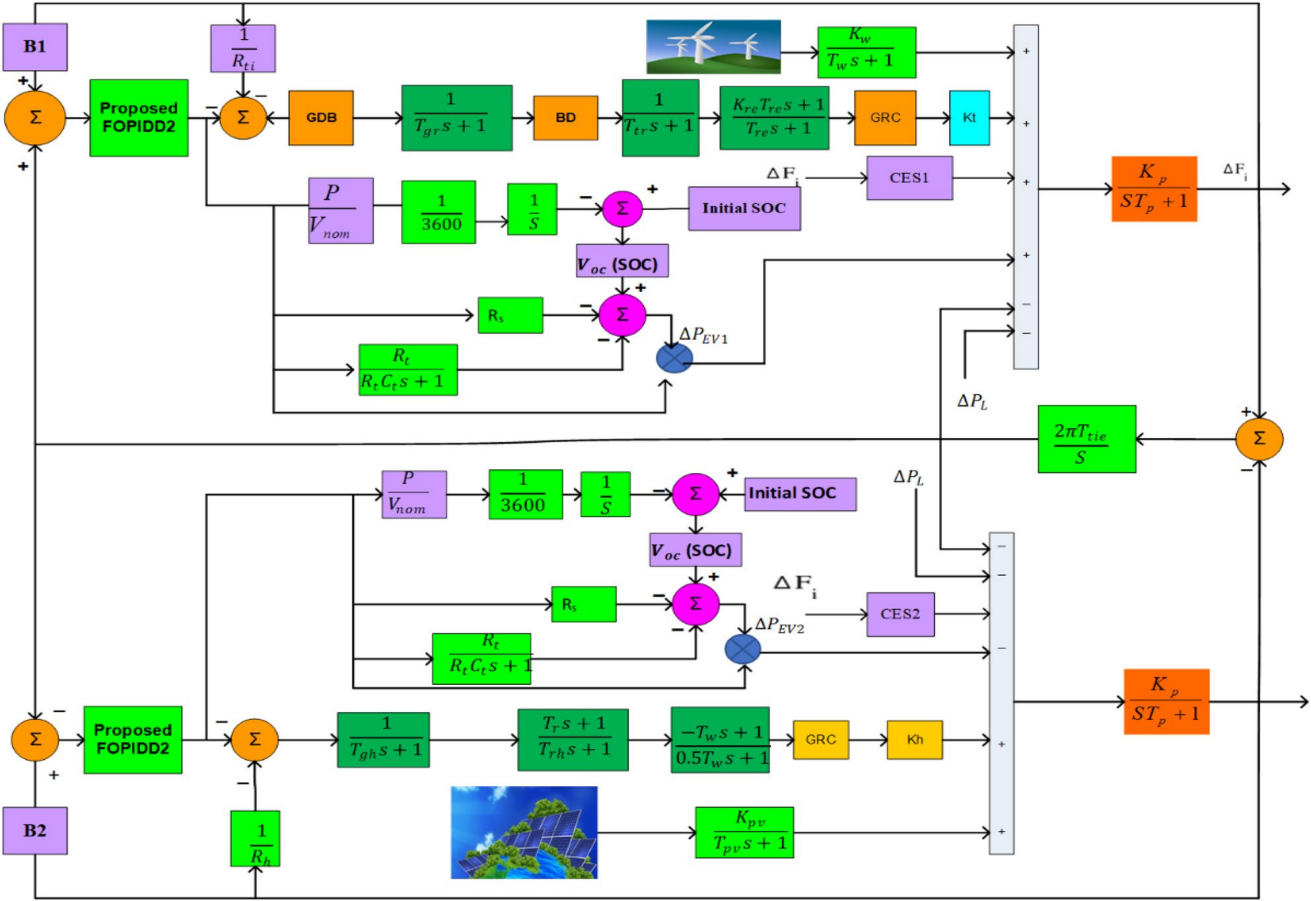
Schematic modelling of proposed HPS.

This section presents a mathematical representation of a combined dual-area power system (PS), which includes renewable energy resources, electric vehicles, capacitor energy storage, and conventional power sources as shown in Fig. 1. The distribution of renewable energy sources (RESs) occurs in distinct zones, where zone 1 comprises solar power and zone 2 comprises wind power. Zone 1 is comprised of a thermal resource, whereas Zone 2 is a hydroelectric structure. It is presumed that the distribution of electric vehicles between the two regions is equivalent. The components used to build the PS system are sourced from^7,47^ and implemented in the Simulink/Matlab environment; additional information is available in Appendix A. The performance of the suggested controllers is significantly influenced by the nonlinearities demonstrated by the components of the system; therefore, it is vital to take these into account during the design and testing stages. The physical constraints of power plants are accounted for by the system under investigation, which includes the governor dead band (GDB) and generation rate constraint (GRC) of thermal units. Both the ascending and decreasing rates have a 10% pu/min (0.0017 pu.MW/s) GRC^28,33^. In addition, the hydropower plant is subject to GRC constraints, which stipulate that the rates of increase and decrease are 270 percent pu/min (0.045 pu.MW/s) and 360 percent pu/min (0.06 pu.MW/s), respectively. Upon linearization, the GDB can be represented by the speed change and its rate of change, resulting in a Fourier series transfer function model that incorporates a 0.5 percent backlash^28,33^. The formulation of this model is as follows:

1 $$ {\text{GDB}} = \frac{{{\text{N}}_{1} + {\text{SN}}_{2} }}{{{\text{T}}_{{{\text{sg}}}} {\text{S}} + 1}} $$

2 $$ {\text{Where N}}_{{1}} = 0.{\text{8 and}},{\text{N}}_{2} = \frac{ - 0.2}{{\uppi }} $$

### Modelling of conventional power systems

Traditional power generation systems consisted of reheat thermal (incorporating a governor-turbine-reheater sub model) and hydropower generation (incorporating a penstock-droop compensation sub model). The subsequent equations illustrate the mathematical representations for reheating thermal systems and their corresponding sub-models, which consist of a governor, turbine, and re-heater^33,48^.

3 $$ G_{G} \left( s \right) = \frac{1}{{\left( {1 + T_{gr} s} \right)}} $$

4 $$ G_{T} \left( s \right) = \frac{1}{{\left( {1 + T_{tr} s} \right)}} $$

5 $$ G_{R} \left( s \right) = \frac{{1 + T_{re} K_{re} s}}{{\left( {1 + T_{re} s} \right)}} $$

6 $$ G_{RT} \left( s \right) = \frac{{1 + T_{re} K_{re} s}}{{\left( {1 + T_{gr} s} \right)\left( {1 + T_{re} s} \right)\left( {1 + T_{tr} s} \right)}} $$

The subsequent sub-models for hydroelectric power, including droop compensation, governor, and penstock, are mathematically represented by the equations listed below^48^.

7 $$ G_{HG} \left( s \right) = \frac{1}{{\left( {1 + T_{h} s} \right)}} $$

8 $$ G_{HT} \left( s \right) = \frac{{\left( {1 + T_{rs} s} \right)}}{{\left( {1 + T_{rh} s} \right)}} $$

9 $$ G_{HD} \left( s \right) = \frac{{\left( {1 - T_{w} s} \right)}}{{\left( {1 + 0.5T_{w} s} \right)}} $$

10 $$ G_{H} \left( s \right) = \frac{{\left( {1 - T_{w} s} \right)\left( {1 + T_{rs} s} \right)}}{{\left( {1 + T_{h} s} \right)\left( {1 + 0.5T_{w} s} \right)\left( {1 + T_{rh} s} \right)}} $$

### Modelling of capacitive energy storage (CES)

Capacitive energy storage (CES) devices are increasingly being incorporated into contemporary power systems for their notable power output and ability to rapidly charge and discharge^49^. An advantage of CES is its capacity to generate an ample amount of electricity in a timely manner in response to increased demand. It is economical, straightforward to operate, and has an extended operational lifespan without sacrificing efficiency. The principal energy storage element within the CES system is a supercapacitor which stores energy in the form of static charge using capacitor plates^50^. CES returns energy that has been stored to the grid during times of high demand. Equation (18) illustrates the variation in the incremental power of CES^51^.

11 $$ {\Delta P}_{CES} = \left[ {\frac{{K_{CES} }}{{1 + sT_{CES} }}} \right]\left[ {\frac{{1 + sT_{1} }}{{1 + sT_{2} }}} \right]\left[ {\frac{{1 + sT_{3} }}{{1 + sT_{4} }}} \right]{\Delta }F $$

The time constants of the two-stage phase compensation blocks are denoted as T1–T4.

### Modelling of electrical vehicles (EVs)

**Figure 2:**
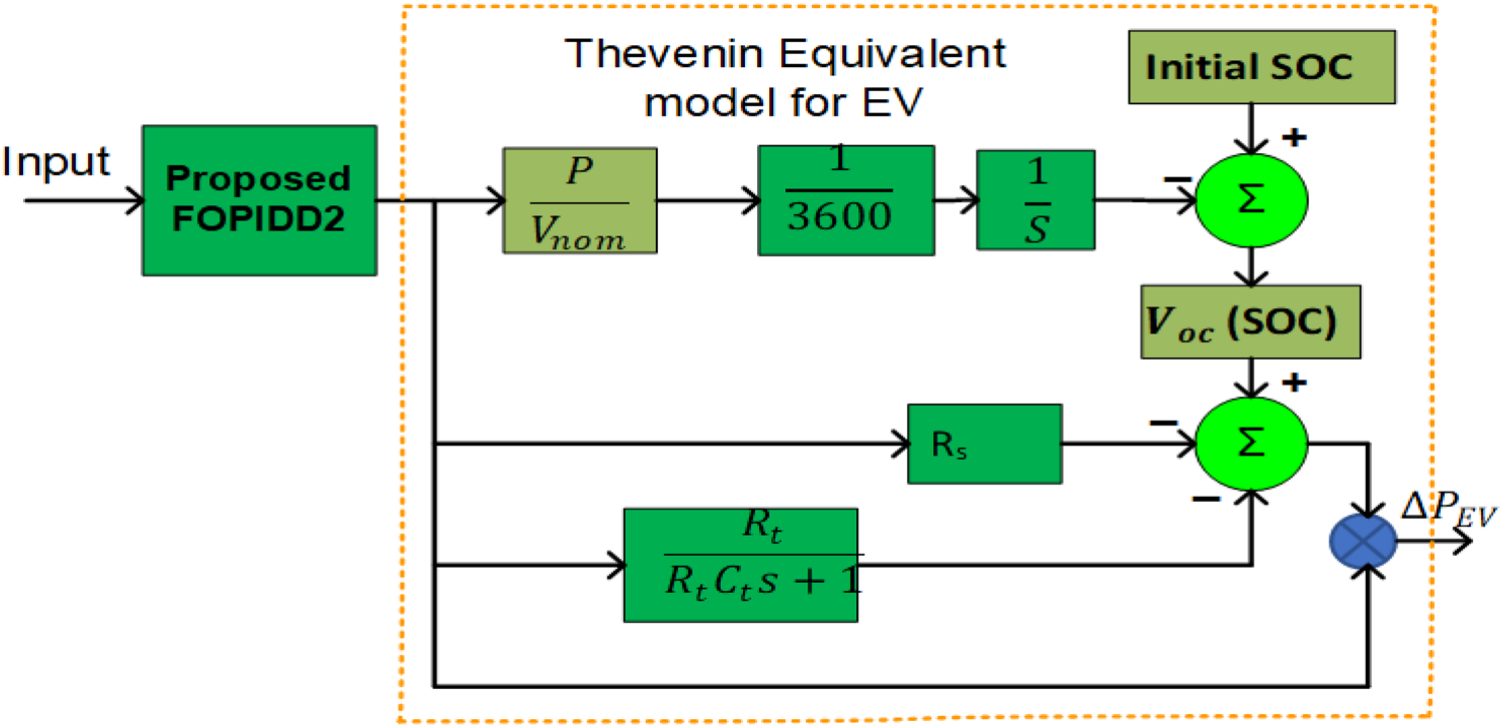
Modeling of EVs for the proposed system.

The recent modification of EVs for regular vehicles in power grids allows for the use of their built-in batteries. Thus, EV batteries with consistent batteries can be regulated to increase frequency adaptability in remote microgrids. EVs also eliminate the need for additional energy storage units in these systems. As a result, EVs can lower system costs and improve the functioning of distant microgrids (MGs). To execute several potential activities, it is necessary to be able to simulate the dynamics of EV energy storage in order to optimize power system sizing, supervision, and control. To express EV functionality in LFC^52,53^, an equivalent Thevenin-based EV representation is used and connected to the dual region power system, as shown in Fig. 2. In this concept, Voc represents the voltage of an open-circuit battery. The voltage of the EVs is ultimately determined by the state of charge (SOC) and voltage of the batteries as shown in below equation^54^.

12 $$ V_{oc} \left( {SOC} \right) = S\frac{RT}{F}{\text{ln}}\left( {\frac{SOC}{{C_{nom} - SOC}}} \right) + V_{nom} $$

where *C*_*nom*_ and *V*_*nom*_ are the nominal contents and batteries voltage powering the EV. The sensitivity parameter is denoted by S, the gas constant is denoted by R, the Faraday constant is denoted by F, and the temperature constant is denoted by T.

### Solar and wind generation modelling

PV service functionality is established by solar radiation and ambient temperature. PV plants’ power output varies with the quantity of sunlight they get. Power electronics-based transfer gadgets are now widely used in PV systems to keep maximum power constant. Injecting the waveforms of high-power-quality currents, they also perform the grid amalgamation function. The execution of power system stability suffers because of fluctuations in output power. The following expression is a model for the power output of solar power plants^55,56^.

13 $$ P = \upeta \varphi_{solar} S\left[ {1 - 0.005\left( {T_{a} + 25} \right)} \right] $$

where $\varphi_{solar}$ stands for solar insolation, $\upeta$ for the PV panel’s conversion efficiency, $T_{a}$ for the ambient temperature, and S for the PV area. In this study, a realistic PV output power has been built to imitate PV inconsistent features based on the design from reference ^57^.

14 $$ G_{PV} \left( s \right) = \frac{{K_{PV} }}{{T_{PV} s + 1}} $$

where $$T_{PV} $$ is the time constant in the PV model and $$K_{PV}$$ is the gain constant.

On the other side, the main factor causing the sporadic characteristics of wind farms is the mechanical wind turbine (WT) power output’s wind speed associated power fluctuating using the below expression^57,58^.

15 $$ P_{wind} = 1/2\rho A_{r} C_{p} V_{\omega }^{3} $$

where $C_{p}$ is the power coefficient, $A_{r}$ is the swept area, $\rho$ is the air density, and $V_{\omega }$ is the wind speed. In this study, a realistic wind output power is created based on the model from^57,58^ to replicate wind erratic features. A model representation of $G_{w} \left( s \right)$ is shown below ^58^.

16 $$ G_{w} \left( s \right) = \frac{{K_{T} }}{{T_{T} s + 1}} $$

where the wind model’s time constant is denoted by $T_{T}$, and its gain constant is denoted by $K_{T}$.

## Squid game optimizer (SGO)

The Squid Game Optimizer (SGO) method, which draws inspiration from the fundamental principles of a traditional Korean game^46^, is presented as a novel metaheuristic algorithm. In the squid game, teams compete to eliminate one another on open fields with no predetermined size restrictions, while attackers strive to attain their objective. The game court, which has a historical cephalopod shape and is approximately half the size of a typical basketball court, functions as the foundation for the mathematical formulation of the algorithm. The algorithm begins by generating its model through the random selection of optimal candidate solutions and an initialization method. The candidates then alternate between two groups of defensive players during a simulated battle. A cost function is utilized to ascertain the champion states of players on opposing factions during the position update procedure. In order to evaluate the performance of the algorithm, twenty-five unrestricted mathematical evaluation functions are applied in conjunction with six prevalent metaheuristics. Furthermore, the efficacy of the SGO is evaluated using real-world scenarios sourced from the most recent CEC (CEC 2020), which unveils remarkable outcomes in addressing intricate optimization issues. The SGO algorithm is comprised of the subsequent stages:

### Mathematical formulation

The mathematical description of the SGO as a metaheuristic method employing the squid game strategy is elaborated in this section. In the first phase, the initialization technique is implemented as follows, considering the seek space to be a distinct region of the field and the potential candidates *(X*_*i*_*)* to be players^46^:

17 $$ X = \left[ \begin{gathered} X_{1} \hfill \\ X_{2} \hfill \\ : \hfill \\ X_{i} \hfill \\ : \hfill \\ X_{n} \hfill \\ \end{gathered} \right] = \left[ \begin{gathered} \begin{array}{*{20}c} \begin{gathered} x_{1}^{1} x_{1}^{2} ....x_{1}^{j} ....x_{1}^{d} \hfill \\ x_{2}^{1} x_{2}^{2} ....x_{2}^{j} ....x_{2}^{d} \hfill \\ \end{gathered} \\ {\begin{array}{*{20}c} \cdots & \ddots & \vdots \\ \end{array} } \\ \end{array} \hfill \\ x_{i}^{1} x_{i}^{2} ....x_{i}^{j} ....x_{i}^{d} \hfill \\ \begin{array}{*{20}c} {\begin{array}{*{20}c} \cdots & \ddots & \vdots \\ \end{array} } \\ {x_{n}^{1} x_{n}^{2} ....x_{n}^{j} ....x_{n}^{d} } \\ \end{array} \hfill \\ \end{gathered} \right],\left\{ \begin{gathered} j = 1,2,.......d \hfill \\ i = 1,2,.......n \hfill \\ \end{gathered} \right. $$

18 $$ x_{i}^{j} = x_{i,\min }^{j} + (x_{i,\max }^{j} - x_{i,\min }^{j} ).rand,\left\{ \begin{gathered} i = 1,2,.......n \hfill \\ j = 1,2,.......d \hfill \\ \end{gathered} \right. $$

where* n* denotes the overall count of participants in the search space, *d* signifies the magnitude of the problem being examined, and the *j*th decision variable or identifies the starting position of the *i*th candidate. The upper and lower limits of the *j*th variable are denoted by $x_{i,\max }^{j}$ and $x_{i,\min }^{j}$ respectively. A random number denoted as “*rand*” is distributed in an even manner from 0 to 1.

19 $$ X^{off} = \left[ \begin{gathered} X_{{_{1} }}^{off} \hfill \\ X_{{_{2} }}^{off} \hfill \\ : \hfill \\ X_{{_{i} }}^{off} \hfill \\ : \hfill \\ X_{m}^{off} \hfill \\ \end{gathered} \right] = \left[ \begin{gathered} \begin{array}{*{20}c} \begin{gathered} x_{1}^{1} x_{1}^{2} ....x_{1}^{j} ....x_{1}^{d} \hfill \\ x_{2}^{1} x_{2}^{2} ....x_{2}^{j} ....x_{2}^{d} \hfill \\ \end{gathered} \\ {\begin{array}{*{20}c} \cdots & \ddots & \vdots \\ \end{array} } \\ \end{array} \hfill \\ x_{i}^{1} x_{i}^{2} ....x_{i}^{j} ....x_{i}^{d} \hfill \\ \begin{array}{*{20}c} {\begin{array}{*{20}c} \cdots & \ddots & \vdots \\ \end{array} } \\ {x_{m}^{1} x_{m}^{2} ....x_{m}^{j} ....x_{m}^{d} } \\ \end{array} \hfill \\ \end{gathered} \right],\left\{ \begin{gathered} i = 1,2,.......m \hfill \\ j = 1,2,.......d \hfill \\ \end{gathered} \right. $$

20 $$ X^{Def} = \left[ \begin{gathered} X_{{_{1} }}^{Def} \hfill \\ X_{{_{2} }}^{Def} \hfill \\ : \hfill \\ X_{{_{i} }}^{Def} \hfill \\ : \hfill \\ X_{m}^{Def} \hfill \\ \end{gathered} \right] = \left[ \begin{gathered} \begin{array}{*{20}c} \begin{gathered} x_{1}^{1} x_{1}^{2} ....x_{1}^{j} ....x_{1}^{d} \hfill \\ x_{2}^{1} x_{2}^{2} ....x_{2}^{j} ....x_{2}^{d} \hfill \\ \end{gathered} \\ {\begin{array}{*{20}c} \cdots & \ddots & \vdots \\ \end{array} } \\ \end{array} \hfill \\ x_{i}^{1} x_{i}^{2} ....x_{i}^{j} ....x_{i}^{d} \hfill \\ \begin{array}{*{20}c} {\begin{array}{*{20}c} \cdots & \ddots & \vdots \\ \end{array} } \\ {x_{m}^{1} x_{m}^{2} ....x_{m}^{j} ....x_{m}^{d} } \\ \end{array} \hfill \\ \end{gathered} \right],\left\{ \begin{gathered} i = 1,2,.......m \hfill \\ j = 1,2,.......d \hfill \\ \end{gathered} \right. $$

where *m* represents the whole participants in every group of games; The* k*th player on defense is = $X_{{_{i} }}^{Def}$ and the *i*th player on offence is = $X_{{_{i} }}^{off}$. At the beginning of the game, one offensive player fights with the defensive players. It is important to note that while defensive players are allowed to utilize both feet, attacking players are only allowed to move and fight with one foot. The mathematical representation of these elements is as follows^46^:

21 $$ DG = \frac{{\sum\limits_{i = 1}^{m} {X_{{_{i} }}^{Def} } }}{m},i = 1,2,.....m $$

22 $$ X_{{_{i} }}^{offNew1} = \frac{{X_{{_{i} }}^{off} + r_{1} \times DG - r_{2} \times X_{r3}^{Def} }}{2},i = 1,2,.....m $$

The offensive players’ capabilities are represented by *r*_*1*_ and *r*_*2*_, which are random values between 0 and 1. The defensive group is represented by (*DG)*, and the *i*th offensive player’s future position on the ground is indicated by $X_{r3}^{Def}$ Є [1 to *m*]. Each player’s fitness function is assessed following a match between the *i*th offensive player and a particular defensive player. The winner is decided by the players’ contest result. Declared the winner, the offensive player becomes a member of the winning offensive group (SOG). In order to achieve this, if the offensive player’s winning status exceeds the defensive player’s, the attacking player may use both feet. These aspects can be expressed mathematically as follows^46^:

23 $$ X^{Sccoff} = \left[ \begin{gathered} X_{{_{1} }}^{Sccoff} \hfill \\ X_{{_{2} }}^{Sccoff} \hfill \\ : \hfill \\ X_{{_{i} }}^{Sccoff} \hfill \\ : \hfill \\ X_{o}^{Sccoff} \hfill \\ \end{gathered} \right] = \left[ \begin{gathered} \begin{array}{*{20}c} \begin{gathered} x_{1}^{1} x_{1}^{2} ....x_{1}^{j} ....x_{1}^{d} \hfill \\ x_{2}^{1} x_{2}^{2} ....x_{2}^{j} ....x_{2}^{d} \hfill \\ \end{gathered} \\ {\begin{array}{*{20}c} \cdots & \ddots & \vdots \\ \end{array} } \\ \end{array} \hfill \\ x_{i}^{1} x_{i}^{2} ....x_{i}^{j} ....x_{i}^{d} \hfill \\ \begin{array}{*{20}c} {\begin{array}{*{20}c} \cdots & \ddots & \vdots \\ \end{array} } \\ {x_{o}^{1} x_{o}^{2} ....x_{o}^{j} ....x_{o}^{d} } \\ \end{array} \hfill \\ \end{gathered} \right],\left\{ \begin{gathered} i = 1,2,.......o \hfill \\ j = 1,2,.......d \hfill \\ \end{gathered} \right. $$

24 $$ SOG = \frac{{\sum\limits_{i = 1}^{o} {X_{{_{i} }}^{Sccoff} } }}{o},i = 1,2,.....o $$

25 $$ X_{{_{i} }}^{offNew2} = X_{{_{i} }}^{offNew1} + r_{1} \times SOG - r_{2} \times BSi,BSi = 1,2,.....m $$

If defensive players’ winning states exceed those of offensive players, the defensive players are declared game winners and invited to join the successful defense group. These defensive players in the group are expected to oversee guarding the bridge, a key feature of the playground. The successful defensive players navigate among the attacking players in the crowd in preparation for a new battle. The following is a mathematical representation of these constituents^46^:

26 $$ SDG = \left[ \begin{gathered} X_{{_{1} }}^{Sccoff} \hfill \\ X_{{_{2} }}^{Sccoff} \hfill \\ : \hfill \\ X_{{_{i} }}^{Sccoff} \hfill \\ : \hfill \\ X_{p}^{Sccoff} \hfill \\ \end{gathered} \right] = \left[ \begin{gathered} \begin{array}{*{20}c} \begin{gathered} x_{1}^{1} x_{1}^{2} ....x_{1}^{j} ....x_{1}^{d} \hfill \\ x_{2}^{1} x_{2}^{2} ....x_{2}^{j} ....x_{2}^{d} \hfill \\ \end{gathered} \\ {\begin{array}{*{20}c} \cdots & \ddots & \vdots \\ \end{array} } \\ \end{array} \hfill \\ x_{i}^{1} x_{i}^{2} ....x_{i}^{j} ....x_{i}^{d} \hfill \\ \begin{array}{*{20}c} {\begin{array}{*{20}c} \cdots & \ddots & \vdots \\ \end{array} } \\ {x_{p}^{1} x_{p}^{2} ....x_{p}^{j} ....x_{p}^{d} } \\ \end{array} \hfill \\ \end{gathered} \right],\left\{ \begin{gathered} i = 1,2,.......p \hfill \\ j = 1,2,.......d \hfill \\ \end{gathered} \right. $$

27 $$ OG = \frac{{\sum\limits_{i = 1}^{m} {X_{{_{i} }}^{off} } }}{m},i = 1,2,.....m $$

28 $$ X_{{_{i} }}^{DefNew1} = \frac{{X_{{_{i} }}^{Def} + r_{1} \times OG - r_{2} \times X_{r3}^{off} }}{2},i = 1,2,.....m $$

An additional step is added to the process as the assaulting players attempt to cross the bridge guarded by the defending units in SDG with the goal of strategically adjusting the exploitation and exploration stages of the predicted algorithm. As a result, all offensive players are authorized to engage in a position-updating operation that directs them towards the most promising candidate solution found thus far as well as a particular defending player in SDG. Below is a mathematical representation of these constituents^46^.

29 $$ X_{{_{i} }}^{offNew1} = \frac{{X_{{_{i} }}^{Scoff} + r_{1} \times BS - r_{2} \times X_{k}^{ScDef} }}{2},\left\{ \begin{gathered} i = 1,2,.....o \hfill \\ k = 1,2,.....p \hfill \\ \end{gathered} \right. $$

**Figure 3:**
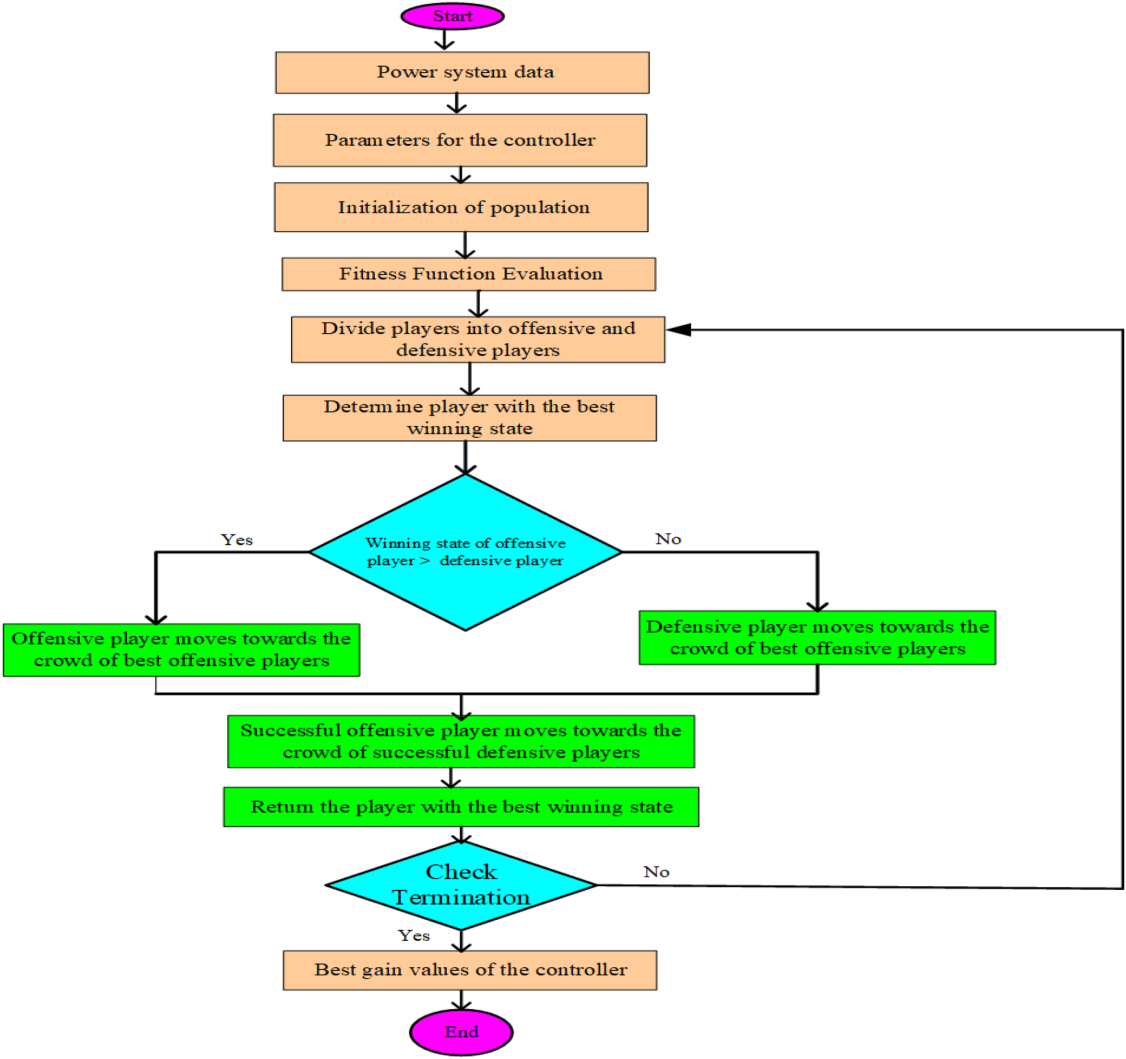
Flow diagram of suggested squid game optimizer.

The best candidate for a solution is represented by *BS* in SOG and SDG, whereas *p* and *o* the total number that represent successful offensive and defensive players, respectively. Figure 3 shows the flowchart for the recommended SGO approach.

## Controller design and formulation of fitness function

**Figure 4:**
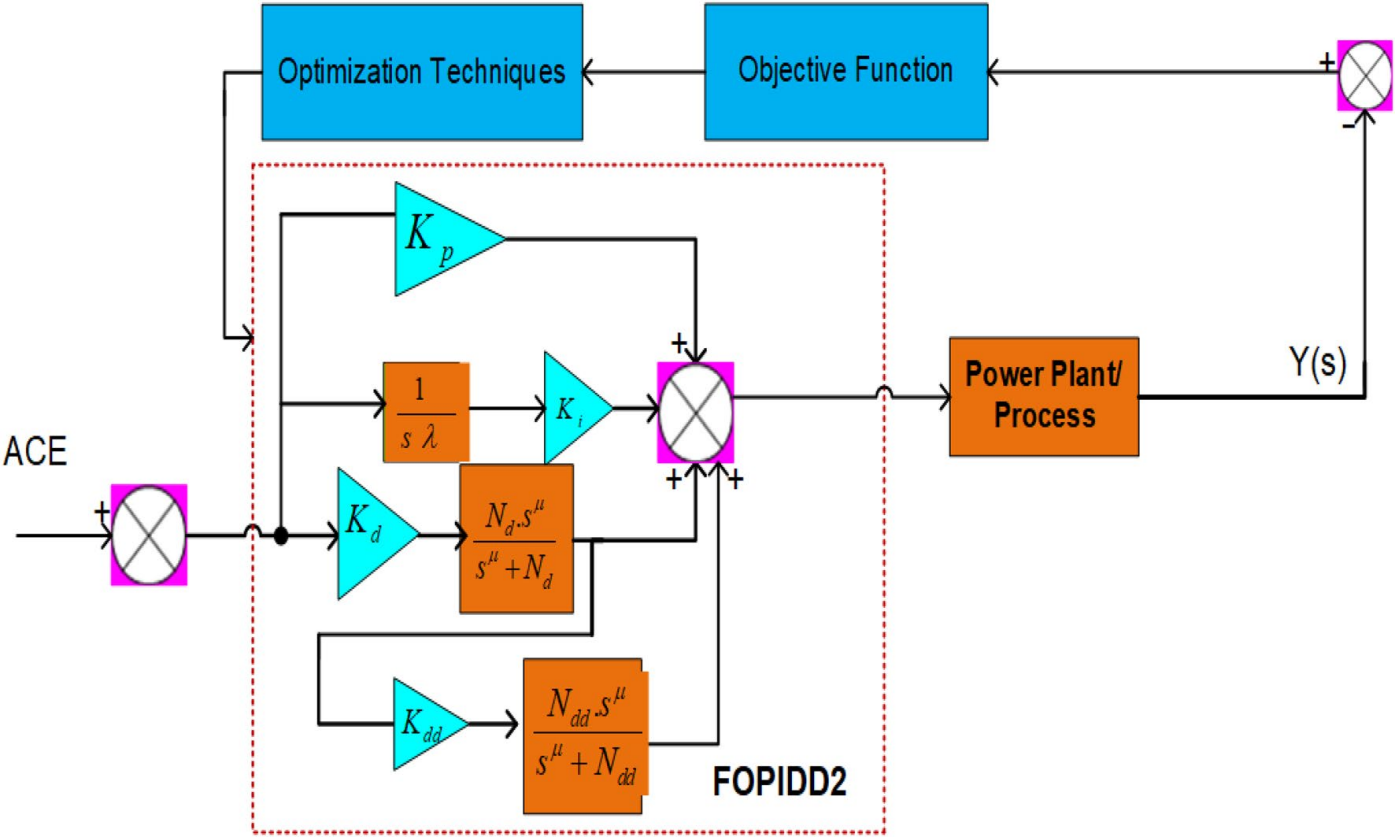
Structure of suggested FOPIDD^2^.

The fundamental goal of redesigning the FOPIDD2 is to improve and regulate the frequency response of a diversified power system dealing with abrupt load changes and fluctuations in renewable energy sources. This controller is suggested for both regions to reduce frequency fluctuations and associated tie-line power imbalances produced by diverse load disturbances and renewable energy variations. Traditional PID controllers, which are widely used in industries due to their simplicity and efficacy, provide the foundation of the PIDD2 structure, which adds a second-order derivative gain, comparable to the normal PID design^59^. Although the FOPIDD2 controller has not received much attention, previous research has shown that both FOPID and PIDD2 controllers outperform typical PID controllers in terms of performance. Figure 4 shows the FOPIDD2 controller’s block diagram, which was constructed by combining the PIDD2 controller and fractional calculus. The FOPIDD2 controller, as opposed to the PIDD2 controller, incorporates the second derivative portion as a fractional order derivative^60,61^. The transfer function of the FOPIDD2 is described in Eq. (30) and the relationship between the system’s control input (U) and the error signal (E) is described in Eq. (31).

30 $$ FOPIDD2 = \frac{Y\left( s \right)}{{R\left( s \right)}} = K_{p} + \frac{{K_{i} }}{{s^{\lambda } }} + K_{d} \left[ {\frac{{N_{d} s^{\mu } }}{{s^{\mu } + N_{d} }}} \right] + K_{d} \left[ {\frac{{N_{d} s^{\mu } }}{{s^{\mu } + N_{d} }}} \right].K_{dd} \left[ {\frac{{N_{dd} s^{\mu } }}{{s^{\mu } + N_{dd} }}} \right] $$

31 $$ {\text{U}}\left( {\text{s}} \right) = E\left( s \right)\left\{ {K_{p} + \frac{{K_{i} }}{{s^{\lambda } }} + K_{d} \left[ {\frac{{N_{d} s^{\mu } }}{{s^{\mu } + N_{d} }}} \right] + K_{d} \left[ {\frac{{N_{d} s^{\mu } }}{{s^{\mu } + N_{d} }}} \right].K_{dd} \left[ {\frac{{N_{dd} s^{\mu } }}{{s^{\mu } + N_{dd} }}} \right]} \right\} $$

where ($N_{d}$, $N_{dd}$) signified the filter terms, ($\lambda ,$ µ) are the integral-differentiator operators, and ($K_{d}$, $K_{p}$, K_i_) signifies the derivative, proportional and integral knobs of the FOPIDD2 controller. The recommended gains for the FOPIDD2 controller were established by minimizing the cost function through the squid game optimizer. The adoption of an ITSE-based cost function^33,40,48^ leads to a reduction in settling time and rapid attenuation of high oscillations.

32 $$ ITSE = J = \mathop \smallint \limits_{0}^{t} t\left[ {\Delta F_{1}^{2} + \Delta F_{2}^{2} + \Delta P_{tie}^{2} } \right]dt $$

33 $$ ITAE = J = \mathop \smallint \limits_{0}^{t} t\left[ {\left| {\Delta F_{1} } \right| + \left| {\Delta F_{2} } \right| + \left| {\Delta P_{tie} } \right|} \right]dt $$

34 $$ ISE = J = \mathop \smallint \limits_{0}^{t} \left[ {\Delta F_{1}^{2} + \Delta F_{2}^{2} + \Delta P_{tie}^{2} } \right]dt $$

FOPIDD2 controller gains are subject to the following restrictions.

35 $$ \begin{gathered} K_{p}^{Min} \le K_{p} \le K_{p}^{Max} ; K_{d}^{Min} \le K_{d} \le K_{d}^{Max} ; \hfill \\ K_{dd}^{Min} \le K_{dd} \le K_{dd}^{Max} ; K_{i}^{Min} \le K_{i} \le K_{i}^{Max} ; \hfill \\ N_{dd}^{Min} \le N_{dd} \le N_{dd}^{Max} , N_{d}^{Min} \le N_{d} \le N_{d}^{Max} , \lambda^{Min} \le \lambda \le \lambda^{Max} ; \hfill \\ \mu^{Min} \le \mu \le \mu^{Max} \hfill \\ \end{gathered} $$

## Results, implementation and discussion

In this portion, the efficacy of the proposed approach is tested in a distinct hybrid power source, along with electric vehicles and capacitor energy storage. The controller knobs are optimized employing the Squid Game Optimizer (SGO) in MATLAB programming and integrated with the Simulink tool for the unified power system. The optimal values for different control algorithms have been depicted in Table 1 after 30 iterations of optimization procedures using data from Appendix B. The proposed FOPIDD2 controller, which employs the SGO approach in conjunction with the EV system, is compared to other controllers such as FOPID, PIDD2, and PID. The outcomes of the scrutinized multi-area Integrated Power System (IPS) are thoroughly evaluated in the following case studies.

**Table 1:** Optimal coefficient values for the suggested approach.

Approach	K_p_	K_I_	K_d_	K_dd_	N_d_	N_dd_	µ	$\lambda$
SGO	3.456	1.900	1.110	2.657	6.090	5.780	0.157	0.234
JSO	5.101	2.546	2.079	8.456	8.567	4.781	0.056	0.767
GWO	1.120	2.109	1.989	3.345	3.300	4.890	0.045	0.458
FOPIDD2	4.789	3.456	3.671	9.675	8.220	7.412	0.052	0.086
FOPID	5.124	3.445	2.089	–	–	–	0.123	0.009
PIDD2	8.124	9.112	3.974	4.897	3.678	1.098	–	–
PID	4.009	1.223	7.009	–	–	–	–	–

### Case-1 (analyses of controller performance)

**Figure 5:**
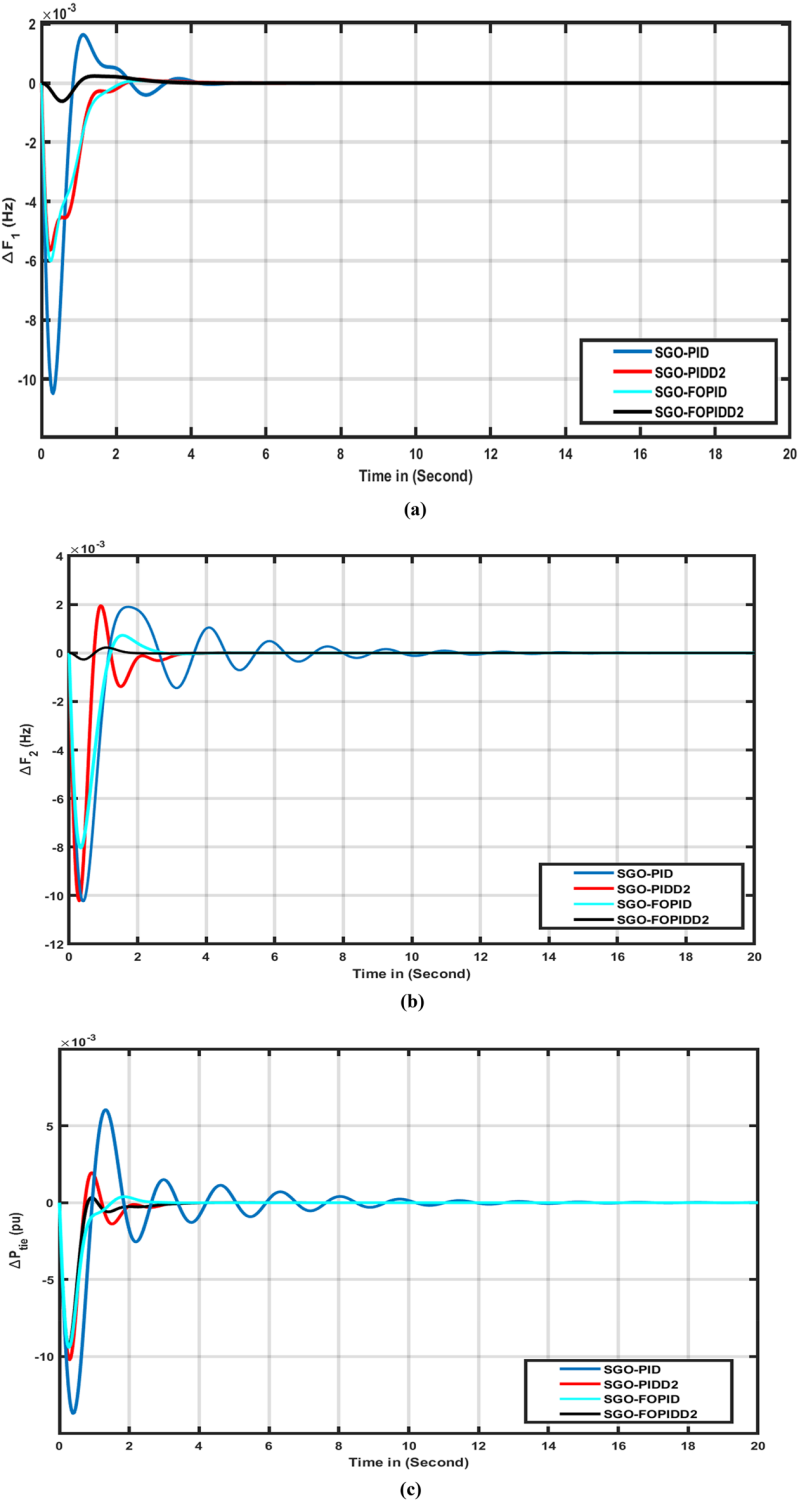
Transient response of HPS with various algorithm techniques in: (**a**) ∆F1 (**b**) ∆F2, (**c**) ∆Ptie.

The effectiveness of the FOPIDD2 was assessed by comparing it with several other controllers including FOPID, PIDD2, PID, and I-TD^45^ in this scenario. The response of each controller was evaluated based on tie line power (ΔPtie), area-2 (ΔF2), and area-1 (ΔF1), as depicted in Fig. 5a-c. Table 2 presents a comprehensive performance analysis of various controllers with respect to transient metrics such as Osh (Overshoot), Ush (Undershoot), and Ts (time settling) for (ΔF2), (ΔPtie), and (ΔF1). The FOPIDD2 control strategy demonstrated faster settling times compared to PID, PIDD2, I-TD^45^, and FOPID controller in regions 1, 2, and the associated tie-line. In comparison to the PID controller, the FOPIDD2 approaches reduced overshoot for (ΔF1), (ΔF2), and (ΔPtie) by 71.66%, 87.98%, and 43.21%, respectively. Furthermore, our proposed approach enhanced the settling time by 21.66%, 19.12%, and 22.09% compared to PID controller. The FOPIDD2 controllers also improved Ts by 19.78%, 12.87%, and 26.09% when compared to the PIDD2 controller, while significantly reducing maximum Osh by 78.98%, and 67.34% for area-2 and tie line, at the cost of decreasing overshoot for area-1 and undershoot by 81.12%, 49.77%, and 08.65% for (ΔF1), (ΔF2), and (ΔPtie). Comparing the FOPIDD2 algorithm to the I-TD^45^ controller, Ts improved by 56.23% for (ΔPtie), 61.34% for (ΔF2), and 49.11% for (ΔF1).

**Table 2:** Comparison performance of several controllers for case 1.

Transient parameters	Variation in areas	I-TD^45^	PID	FOPID	PIDD2	FOPID
Time settling	∆F1	12.27	4.01	2.83	3.29	2.22
	∆F2	29.46	11.93	2.64	3.23	1.52
	∆Ptie	30.50	12.11	3.83	3.66	2.81
Overshoot	∆F1	0.00280	0.001632	0.0001233	0.0000768	0.00023470
	∆F2	0.00110	0.001890	0.0007254	0.0019490	0.0002201
	∆Ptie	0.0007	0.006044	0.0017100	0.0019490	0.0003508
Undershoot	∆F1	− 0.0109	− 0.010480	− 0.006548	− 0.006016	− 0.0006158
	∆F2	− 0.0035	− 0.010240	− 0.008038	− 0.010220	− 0.0002686
	∆Ptie	− 0.0022	− 0.013700	− 0.010490	− 0.010220	− 0.009405

### Case-2 (analyses of algorithm performance)

**Figure 6:**
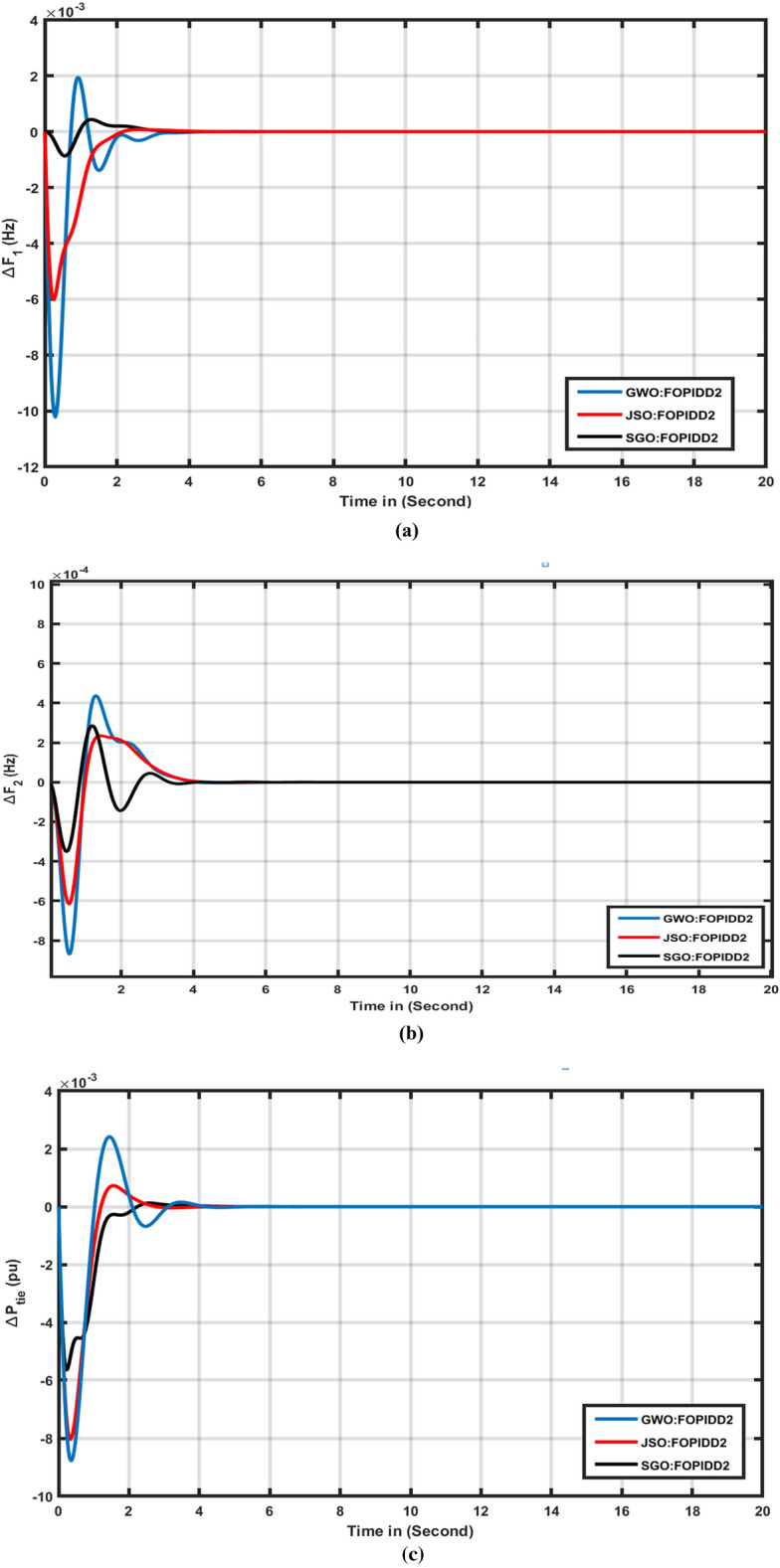
Transient response of HPS with various algorithm techniques in: (**a**) ∆F1 (**b**) ∆F2, (**c**) ∆Ptie.

**Figure 7:**
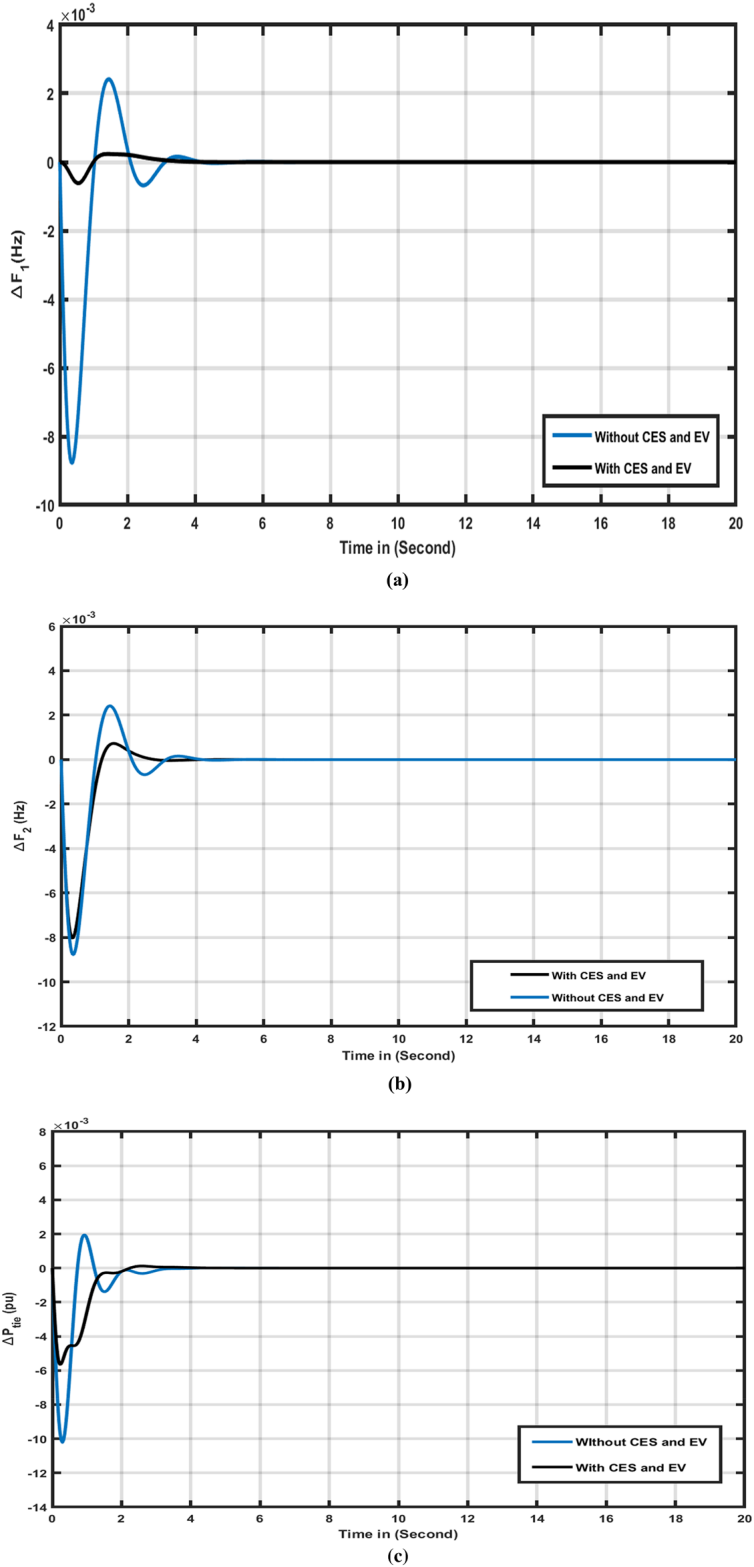
Transient response of HPS with various algorithm techniques in: (**a**) ∆F1 (**b**) ∆F2, (**c**) ∆Ptie.

This study compared the efficacy of the squid game optimizer (SGO) with various modern algorithms including the jellyfish swarm optimization (JSO), Grey wolf optimizer (GWO), Water Cycle Algorithm (WCA)^45^ and Path Finder Algorithm (PFA)^43^. The response of each algorithm was evaluated based on tie line (ΔPtie), area-2 (ΔF2), and area-1 (ΔF1), as depicted in Fig. 6a-c. Table 3 presents a comprehensive performance analysis of various algorithms with respect to transient metrics such as O_sh_, U_sh_, and T_s_ for (ΔPtie), (ΔF1), and (ΔF2). From Table 3 and Fig. 7a-c it can be observed that SGO: FOPIDD2 have better settling times as compared to JSO, GWO, WCA^45^, and FPA metaheuristic algorithms in regions 1, 2, and the associated tie-line. In comparison to the JSO algorithm, the SGO approaches reduced overshoot for (ΔPtie), (ΔF1), and (ΔF2) by 27.20%, 35.11%, and 23. 21%, respectively. Furthermore, our proposed approach enhanced the settling time by 34.11%, 13.43%, and 29.88% compared to grey wolf optimizer algorithms. The SGO algorithms also improved Ts by 13.98%, 47.67%, and 54.54% when compared to the jellyfish search algorithm, while significantly reducing maximum Osh by 87.09%, 81.12%, and 76.78%, and undershoot by 81.19%, 66.54%, and 93.76% for (ΔPtie), (ΔF1), and (ΔF2). Our proposed SGO: FOPIDD2 algorithm also performed very well as compared to WCA: I-TD^45^ and FPA: FOTID^43^ approaches in respect of enhanced settling time, minimum overshoot and undershoot to the WCA: ITD optimizer algorithm for (ΔPtie), area-2 (ΔF2), and area-1 (ΔF1).

**Table 3:** Comparison performance of several algorithms for scenario 2.

Transient parameters	Variation in areas	JSO: FOPIDD2	GWO: FOPIDD2	SGO: FOPIDD2	WCA: I-TD^45^	PFA: FOTID^43^
Time settling (Ts)	∆F_1_	3.66	3.80	2.86	12.27	25.5
	∆F_2_	4.09	4.85	3.93	29.46	23.2
	∆P_tie_	3.71	3.83	3.66	30.50	18.77
Overshoot (O_sh_)	∆F_1_	0.00007	0.00194	0.00043	0.00280	0.00680
	∆F_2_	0.00024	0.00044	0.00028	0.00110	0.01170
	∆P_tie_	0.00072	0.00241	0.00012	0.0007	0.00260
Undershoot (U_sh_)	∆F_1_	− 0.00600	− 0.0102	− 0.00086	− 0.0109	− 0.0245
	∆F_2_	− 0.00062	− 0.0008	− 0.00035	− 0.0035	− 0.0228
	∆P_tie_	− 0.00803	− 0.0087	− 0.00564	− 0.0022	− 0.0044

### Case-3 (Analysis of electrical vehicles and capacitive energy storage)

Scenario 3 assesses the outcomes of integrating Capacitor Energy Storage (CES) and Electric Vehicles (EVs) into an existing hybrid energy system. The effectiveness of the system is measured using the Squid Game Optimizer (SGO)-based FOPIDD2 controller, both with and without the effects of EVs and CES. Results for tie line (ΔPtie), area-2 (ΔF2), and area-1 (ΔF1) are depicted in Fig. 7a–c and summarized in Table 4. The Figures illustrate that our proposed SGO-FOPIDD2, incorporating CES and EVs, outperforms in terms of reduced oscillation, undershoot, and overshoot for area-1 (Ts = 2.88, Osh = 0.002347, Ush =  − 0.0006158), Area-2 (Ts = 2.60, Osh = 0.00072454, Ush =  − 0.0008038), and tie-line (Ts = 2.51, Osh = 0.000120, Ush =  − 0.0056199) compared to the SGO-FOPIDD2 without CES and EV effects for Area1 (Ts = 4.00, Osh = 0.0024142, Ush =  − 0.008772), Tie-line (Ts = 3.65, Osh = 0.0019389, Ush =  − 0.0102192) and Area-2 (Ts = 4.33, Osh = 0.0024140, Ush =  − 0.008771), Fig. 7a-c indicates that the system’s response to EVs and CES unit effects yields better outcomes in terms of Osh, Ts, and Ush for (∆F1), (∆F2), and (∆Ptie) compared to the system’s response without EVs and CES unit effects. Table 4 further underscores the remarkable results achieved by combining our proposed technique with EVs and CES.

**Table 4:** Comparison performance for case 3.

Transient parameters	System consideration	Without CES and EV	With CES and EVS
Time settling (Ts)	∆F_1_	4.00	2.88
	∆F_2_	4.33	2.60
	∆P_tie_	3.65	2.51
Overshoot (O_sh_)	∆F_1_	0.0024142	0.0002347
	∆F_2_	0.0024140	0.0007254
	∆P_tie_	0.0019389	0.0001220
Undershoot (U_sh_)	∆F_1_	− 0.008772	− 0.0006158
	∆F_2_	− 0.008771	− 0.008038
	∆P_tie_	− 0.0102192	− 0.0056199

### Case-4 (Sensitivity analysis/robustness)

**Figure 8:**
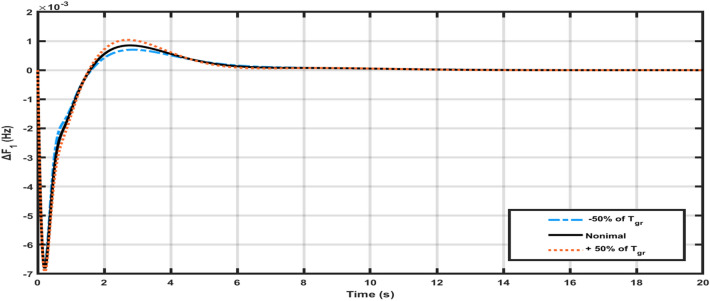
Variation of T_gr_ power system parameters for ∆F1.

**Figure 9:**
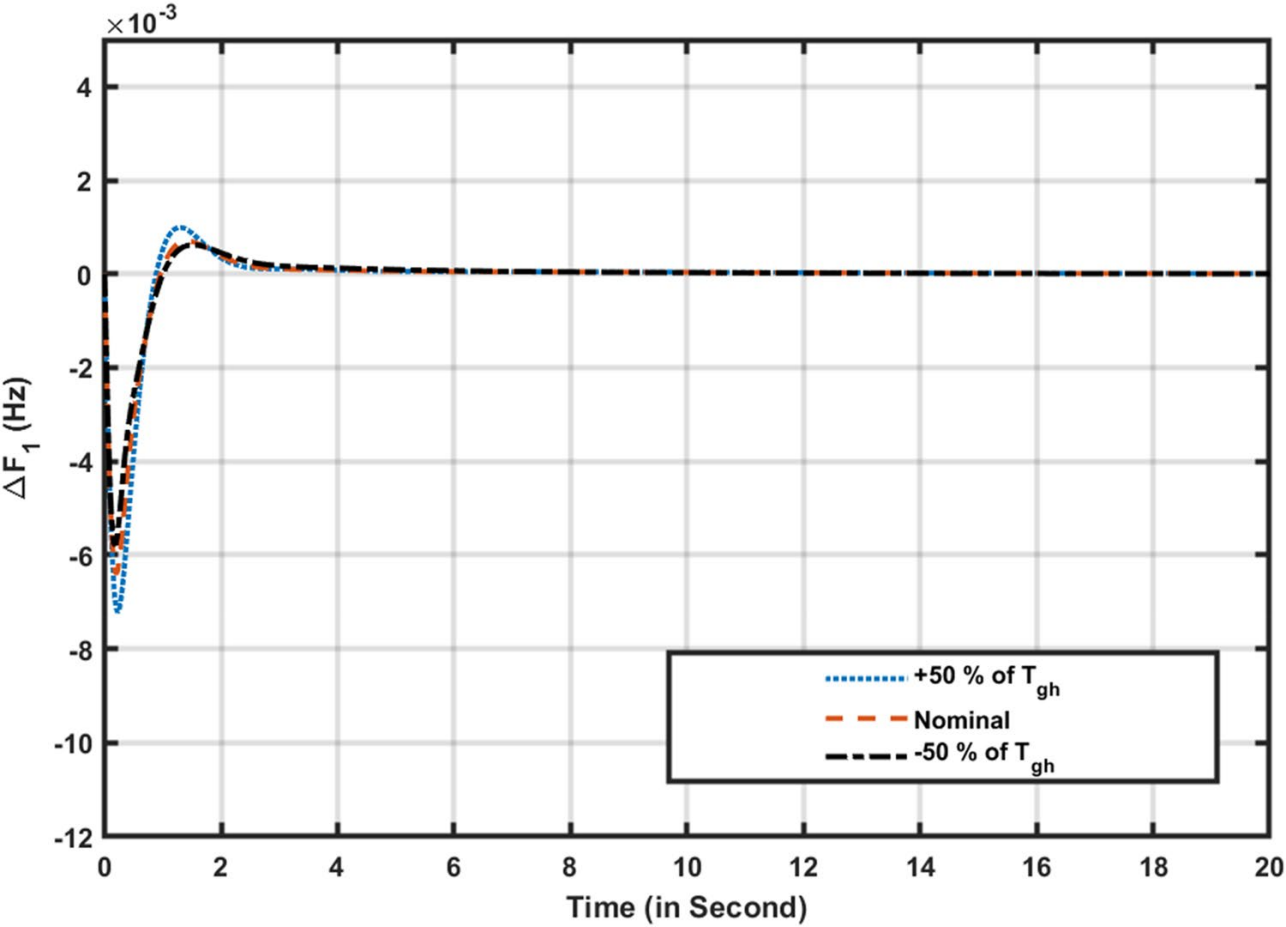
Variation of T_gh_ power system parameters for ∆F1.

**Figure 10:**
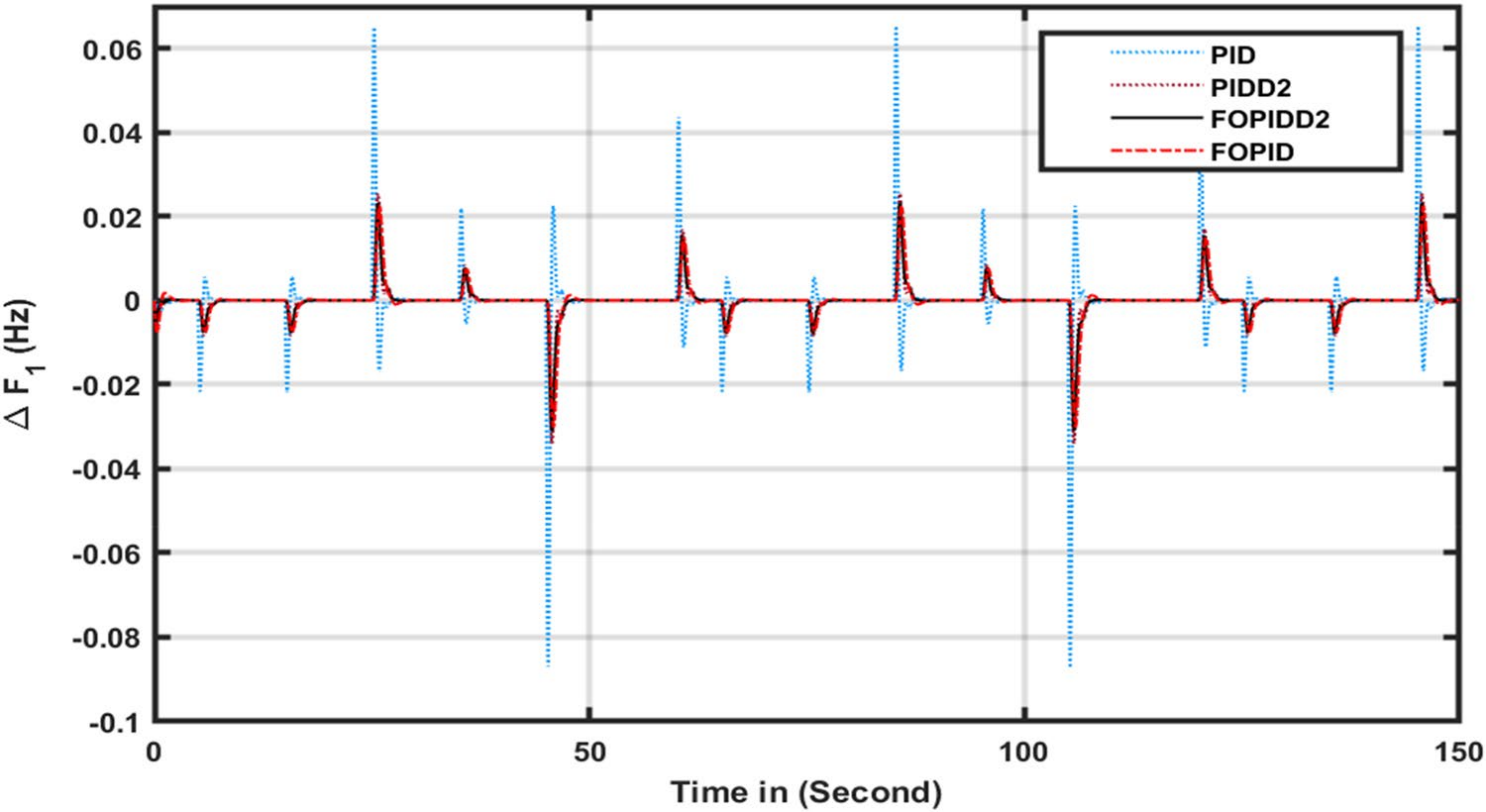
Random load variations for hybrid power systems.

A sensitivity analysis was performed to evaluate the robustness of the optimized FOPIDD2 controller recommended by the Squid Game Optimizer (SGO). The system’s stability may be compromised if the suggested control mechanism fails to adequately adjust to variations in system parameters. In order to assess the stability of the proposed controller, different metrics for parameters such as Tgr, Tgh, and Kw have been modified by about ± 50% and compared to their original parameter responses. Figures 8, 9 illustrate the reliability of the proposed controller by varying the system parameters of the hybrid power systems. The parameters in Table 5, nearly match their nominal values, indicating that the proposed SGO-FOPIDD2 controller consistently performs well within a range of around ± 50% of the system’s characteristics. Moreover, the optimal values of the suggested controller avoid the necessity of resetting when implemented with the real values at the specified value throughout a broad spectrum of parameters. Figure 10 represents the random load variation for the hybrid power systems and indicates that SGO-FOPIDD2 controllers performs excellent as compared to other controllers having a smaller number of oscillations.

**Table 5:** Variations in hybrid power system parameters.

Transient contents	Parameters of power system	% change	∆F1	∆F2	∆P_tie_
Overshoot	T_gr_	+ 50**%**	0.001040	0.00430	0.000123
		− 50**%**	0.001060	0.000440	0.000129
		Nominal	0.001020	0.000420	0.000122
	T_gh_	+ 50**%**	0.0002201	0.0003407	0.0007254
		− 50**%**	0.0002226	0.0003467	0.0007294
		Nominal	0.0002105	0.0003458	0.0007798
	K_w_	+ 50**%**	0.0003708	0.0003834	0.0017100
		− 50**%**	0.0003669	0.0003785	0.0017600
		Nominal	0.0003669	0.0003809	0.0017340
Undershoot	T_gr_	+ 50**%**	− 0.006741	− 0.0008692	− 0.006016
		− 50**%**	− 0.006720	− 0.000884	− 0.006001
	T_gh_	Nominal	− 0.000268	− 0.000351	− 0.000622
		+ 50**%**	− 0.000220	− 0.000392	− 0.000583
		− 50**%**	− 0.000223	− 0.000367	− 0.000603
	K_w_	− 50**%**	− 0.006016	− 0.005648	− 0.007830
		Nominal	− 0.006080	− 0.005490	− 0.008090
		+ 50**%**	− 0.006267	− 0.0005878	− 0.008689
Time settling	T_gr_	+ 50**%**	6.12	6.85	6.89
		− 50**%**	6.61	6.93	6. 87
		Nominal	6.10	6.80	6.84
	T_gh_	+ 50**%**	3.20	3.66	3.66
		− 50**%**	3.22	3.56	3.62
		Nominal	3.18	3.60	3.60
	K_w_	Nominal	4.52	3.64	4.78
		− 50%	4.81	3.63	4.80
		+ 50%	4.88	3.66	4.82

## Conclusion and future study

This article introduces the SGO-based FOPIDD2 methodology as an improvement for Load Frequency Control (LFC) problems in various two-area power systems that include solar, wind, hydro, reheat thermal, electric vehicles, and capacitive energy storage. The superiority of the SGO-based FOPIDD2 controller is proven by a comparison assessment employing recent metaheuristic algorithms as well as different control methodologies. The SGO-based FOPIDD2 approach exhibits superior performance compared to GWO based FOPIDD2, JSO based FOPIDD2, and WCA based I-TD controllers in respect of settling times, and peak under/overshoots. The FOPIDD2 controllers improved Ts by 19.78%, 12.87%, and 26.09% when compared to the PIDD2 controller. In the same manner, the SGO algorithms also improved time settling by 13.98%, 47.67%, and 54.54% when compared to the jellyfish search algorithm, while significantly reducing maximum O_sh_ by 87.09%, 81.12%, and 76.78%, and U_sh_ by 81.19%, 66.54%, and 93.76% for (ΔPtie), (ΔF1), and (ΔF2). Furthermore, the results reveal that SGO based FOPIDD2 superiorly perform with the integration of capacitive energy storages and electrical vehicles in respect of improved settling time, decrease overshoot and undershoot values as compared to without including the effecting of energy storage unit. The recommended SGO-FOPIDD2 has been found to be resilient and exhibits exceptional performance when faced with different sizes of load disturbances and variations in system components. In future, the proposed work may be further enhanced by incorporating with an additional inertial system and can be employed with some recent and advanced optimization techniques.

### Supplementary Information

Supplementary Information.

## Data Availability

All data generated or analyzed during this study are included in this published article [and its supplementary information files].
