# Supplementary material for: Load frequency stabilization of distinct hybrid conventional and renewable power systems incorporated with electrical vehicles and capacitive energy storage
Source: Sci Rep. 2024 Apr 24;14:9400. doi: 10.1038/s41598-024-60028-3 (PMC11043365; doi:10.1038/s41598-024-60028-3)
Supplement: Supplementary file 1 — Supplementary Information. [file 41598_2024_60028_MOESM1_ESM.docx]

**Appendix A [7, 26, 49, 55]**

| **Parameters and their values for EVs** | | | |
| --- | --- | --- | --- |
| V_nom_ | V_nom_ | V_nom_ | V_nom_ |
| R_s_ | R_s_ | R_s_ | R_s_ |
| C_t_ | C_t_ | C_t_ | C_t_ |
| Minimum SOC (in Percentage) | Minimum SOC (in Percentage) | Minimum SOC (in Percentage) | Minimum SOC (in Percentage) |
| C_Batt_ | C_Batt_ | C_Batt_ | C_Batt_ |
| **LFC** | | | |
| T_ps1_ | 11.49 | (K_ps1_) | 68.97 |
| R_H_ | 2.4 | (K_ps2_) | 68.97 |
| T_ps2_ | 11.49 | B2 | 0.4312 |
| R_T_ | 2.4 | B1 | 2.4 |
| **Renewable energy resources** | **LFC model** | **LFC model** | **LFC model** |
| Ks | 0.5 | K_T_ | 1 |
| Ts | 1 | T_T_ | 0.3 |
| K_WTG_ | 1 | T_WTG_ | 1.5 |
| **Hydro Power System** | | | |
| T_w_ | 1 | T_rh_ | 28.749 |
| Kh | 0.32586 | Tr | 5 |
| T_gh_ | 0.2 |  |  |
| **Renewable energy resources** | | | |
| Ks | 0.5 | K_T_ | 1 |
| Ts | 1 | T_T_ | 0.3 |
| K_WTG_ | 1 | T_WTG_ | 1.5 |
| **Boiler Dynamic** | | | |
| *Cb* | 200 | K3 | 0.92 |
| (Trb) | 0.545 | (Tf) | 0.23 |
| (Tr) | 1.4 | (T_rh_) | 28.75 |
| (K1) | 0.85 | (K2) | 0.095 |
| (T_1b_) | 0.545 | (K_1b_) | 0.950 |

| **Parameters** | **Values** | | | **parameters** | **Values** | |  |
| --- | --- | --- | --- | --- | --- | --- | --- |
| Population umber  Lower Limit | | 30  -10 | | Iteration  Upper Limit | 80  10 | |  |
| No of dimension | | 8 | Random numbers | | | [0, 1] | |

Appendix B: SGO parameters:
